# Characteristics and Prognostic Nomogram for Primary Lung Lepidic Adenocarcinoma

**DOI:** 10.1155/2022/3676547

**Published:** 2022-08-31

**Authors:** Hui Tang, Caixia Qiao, Yingyi Wang, Chunmei Bai

**Affiliations:** ^1^Department of Medical Oncology, Peking Union Medical College Hospital, Chinese Academy of Medical Sciences and Peking Union Medical College, Beijing, China; ^2^Department of Medical Oncology, Liaocheng Third People's Hospital, Liaocheng, China

## Abstract

**Background:**

Lepidic adenocarcinoma (LPA) is an infrequent subtype of invasive pulmonary adenocarcinoma (ADC). However, the clinicopathological features and prognostic factors of LPA have not been elucidated.

**Methods:**

Data from the Surveillance, Epidemiology, and End Results (SEER) database of 4191 LPA patients were retrospectively analyzed and compared with non-LPA pulmonary ADC to explore the clinicopathological and prognosis features of LPA. Univariate and multivariate Cox proportional hazard models were performed to identify independent survival predictors for further nomogram development. The nomograms were validated using the concordance index, receiver operating characteristic curves, and calibration plots, as well as decision curve analysis, in both the training and validation cohorts.

**Results:**

Compared with non-LPA pulmonary ADC patients, those with LPA exhibited unique clinicopathological features, including more elderly and female patients, smaller tumor size, less pleural invasion, and lower histological grade and stage. Multivariate analyses showed that age, sex, race, tumor location, primary tumor size, pleural invasion, histological grade, stage, primary tumor surgery, and chemotherapy were independently associated with overall survival (OS) and cancer-specific survival (CSS) in patients with LPA. The nomograms showed good accuracy compared with the actual observed results and demonstrated improved prognostic capacity compared with the TNM stage.

**Conclusions:**

LPA is more frequently diagnosed in older people and women. LPA was inclined to be smaller in tumor size and lower in tumor grade and staging, which may indicate a favorable prognosis. The constructed nomograms accurately predict the long-term survival of LPA patients.

## 1. Introduction

Lung cancer is the leading cause of cancer-related death and one of the most commonly diagnosed cancers worldwide [1]. Lepidic adenocarcinoma (LPA), also known as lepidic predominant adenocarcinoma or nonmucinous bronchioloalveolar carcinoma [2], is an infrequent subtype of lung adenocarcinoma (ADC) without precise incidence data. LPA is defined as an ADC of >3 cm in tumor size and/or has >5 mm lymphatic, vascular, or pleural invasion with a nonmucinous lepidic predominant growth pattern [3]. The definition was proposed by the International Association for the Study of Lung Cancer in 2011 and subsequently accepted by the World Health Organization (WHO) in 2015 [4]. LPA exhibits unique clinicopathological features, specific gene mutation profiles, and desirable survival outcomes compared with lung adenocarcinoma, not otherwise specified (NOS) [4–6]. However, very few population-based studies have been completed on the analysis of the demographic and clinicopathological features and the factors influencing the prognosis of LPA. Meanwhile, it is quite challenging for clinicians to accurately predict the prognosis of patients relying only on tumor-node-metastasis (TNM) stage. Therefore, it is necessary to develop tools for estimating the probability of long-term survival in patients with LPA.

The Surveillance, Epidemiology, and End Results (SEER) database provides a wide range of demographic, clinical, and follow-up information of cancer patients, which was established in 1973 and covers approximately 28% of the population in the United States [7]. Using the SEER database, we retrospectively analyzed the clinicopathological features and survival data of 4191 LPA patients to confirm their clinicopathological characteristics and prognostic indicators. We then developed nomograms estimating the overall survival (OS) and cancer-specific survival (CSS) of LPA patients. Furthermore, we performed nomogram validation in both the training and validation cohorts, as well as decision curve analysis (DCA), to evaluate the accuracy of the nomograms. In addition, we estimated the incidence of LPA and explored the risk factors associated with distant and lymph node metastases of LPA.

## 2. Methods

### 2.1. Data Source and Selection

Patient data were obtained from the SEER database using SEER^*∗*^ Stat software, version 8.4.0.1 (https://seer.cancer.gov/seerstat/). Lung adenocarcinoma was classified according to the 2021 WHO classification system. The International Classification of Diseases for Oncology, third edition (ICD-O-3) histology code, was used in this study to identify patients. The inclusion criteria were as follows: (1) primary lung cancer; (2) ICD-O-3 histology code 8250/3 (lepidic adenocarcinoma), 8260/3 (papillary adenocarcinoma), 8230/3 (solid adenocarcinoma), or 8140/3 (adenocarcinoma-NOS); (3) positive histological confirmation; and (4) diagnosis between 2005 and 2016 to ensure a minimum follow-up period of three years. The exclusion criteria were as follows: (1) patients who had multiple primary tumors in their lifetime; (2) unknown survival data or TNM stage; and (3) unknown important and easily accessible information in clinical practice, including age at diagnosis, race, and marital status. To construct and validate the nomograms, the patients with LPA diagnosed in 2010 and 2011 (*n* = 833) were assigned to the validation cohort, and those diagnosed between 2005 and 2016, except for 2010 and 2011 (*n* = 3358), were assigned to the training cohort.

### 2.2. Study Variables

Demographic and clinicopathological variables of the included patients were extracted, including age, sex, race, marital status, tumor location, primary tumor size, separate tumor nodules, pleural invasion, histological grade, 6th edition TNM stage, treatment, vital status, survival time, corresponding death causes, and the status of education and income in the county where patients resided in. In this study, other races were recorded as “Others,” except for White and Black races. “Married (including common law)” was recorded as “Married,” and other marital statuses were recorded as “Single.” The status of education and income was defined as “Low” or “High,” meaning that patients resided in counties with lower/higher education or income than the median level. Considering that the survival time in the SEER database was expressed in months, the survival time of 0 month was recorded as 0.5 month. OS was defined as the period from diagnosis to death caused by any cause or the last follow-up, while CSS was defined as the period from diagnosis to death caused by lung cancer.

### 2.3. Statistical Analysis

For descriptive statistics, the absolute number and percentage of variables were described. The chi-square tests were used to compare the demographic and clinicopathological characteristics among different groups. Propensity score matching (PSM) analysis was used to minimize the impact of confounding factors. The propensity score for each patient with ADC-NOS or LPA was calculated with a logistic regression model, which included the following variables: age, sex, race, marital status, income and education levels, primary tumor location and size, separate tumor nodules, pleural invasion, histological grade, TNM stage, and treatment. Caliper matching within a caliper of 0.02 was performed among the two groups. After PSM analysis, 4165 pairs of patients were successfully matched among the patients included in our study. OS and CSS were compared between matched patients with ADC-NOS and LPA by the Kaplan–Meier curves and log-rank tests. Then, the LPA patient data were used for further analyses. Multivariate binary logistic regression analyses were performed to identify risk factors for distant and lymph node metastases in all LPA patients. Univariate and multivariate Cox proportional hazard models with a backward stepwise selection method were performed to calculate the hazard ratios (HRs) with 95% confidence intervals (CIs) of variables associated with OS and CSS in the training cohort of LPA patients. Based on multivariate Cox analyses, nomograms were constructed and evaluated by the concordance index (C-index), receiver operating characteristic (ROC) curves, and calibration curves, which were used for the comparison between the observed and nomogram-predicted survival outcomes. Ultimately, decision curve analysis (DCA) was performed to compare the prognostic capacity of the nomogram model and TNM stage. To verify the applicability of the nomogram model, nomograms were validated in both the training and validation cohorts.

The ages of patients were stratified by the X-tile program (Yale University, USA) [8]. According to the desirable cutoff value of age, in terms of OS, determined by X-tile analysis (Supplementary Figures S1A–S1C), the patients were divided into 3 groups (0–69, 70–79, and 80+ years old). All statistical analyses were performed using *R* version 3.6.1 (https://www.r-project.org/). A two-tailed value of *P* < 0.05 was considered to be statistically significant.

## 3. Results

### 3.1. Patients and Tumor Characteristics

Among 1,244,493 patients diagnosed with a primary lung or bronchus malignancy in the SEER database between 1975 and 2016, a total of 27,142 patients were diagnosed with LPA, which accounted for 2.18% of all lung cancer patients. After applying the inclusion and exclusion criteria, the numbers of patients with lung ADC-NOS, LPA, papillary adenocarcinoma, and solid adenocarcinoma enrolled in our study was 95004, 4191, 1545, and 163, respectively. The demographic and clinicopathological characteristics of the eligible patients are shown in Table 1, Supplementary Tables S1 and S2. Among the eligible patients, those with LPA were more common in older age, females, and Yellow race. In addition, patients with LPA were inclined to have smaller tumor sizes, fewer separate tumor nodules, less pleural invasion, and lower histological grades and stages. After PSM analysis, the Kaplan–Meier curves and log-rank tests were performed and showed that patients with LPA had better survival outcomes than those with ADC-NOS (Supplementary Figures S2A–S2B). Furthermore, the survival outcomes were also compared between patients with LPA and papillary adenocarcinoma, or solid adenocarcinoma (Supplementary Figures S3A-S3B, Figures S4A-S4B).

### 3.2. Factors Associated with Distant and Lymph Node Metastases

As shown in Supplementary Table S3, the factors significantly associated with distant metastasis were identified by univariate Cox regression and further examined by multivariate analysis, which showed that Yellow race, large tumor size, positive separate tumor nodules, and higher histological grade were independent risk factors for distant metastasis. Moreover, age, sex, race, tumor size, pleural invasion, and histological grade were significantly associated with lymph node metastasis in the multivariate analysis (Supplementary Table S4).

### 3.3. Establishment of the Nomograms Predicting OS and CSS of LPA Patients

**Figure 1:**
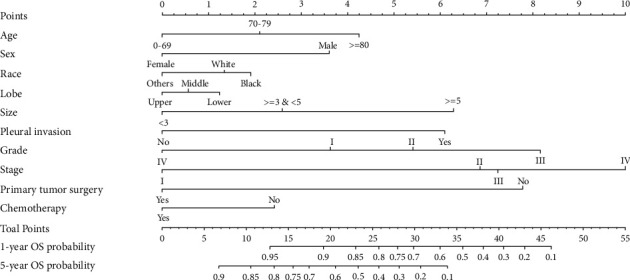
Nomogram predicting the survival probability of 1- and 5-year overall survival in patients with lepidic adenocarcinoma.

**Figure 2:**
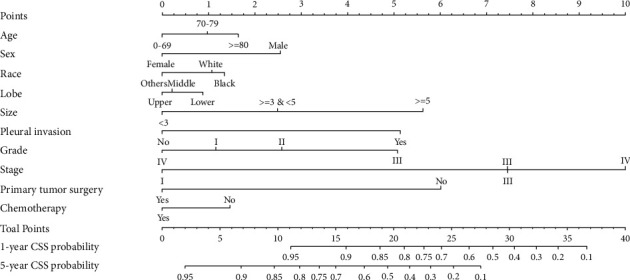
Nomogram predicting the survival probability of 1- and 5-year cancer-specific survival in patients with lepidic adenocarcinoma.

As described above, patients with LPA were divided into a training cohort and a validation cohort. Demographic and clinicopathological characteristics in the two cohorts were overall comparable (Supplementary Table S5). In the training cohort, univariate analysis showed that age, sex, race, marital status, education, tumor location, primary tumor size, separate tumor nodule, pleural invasion, histological grade, TNM stage, primary tumor surgery, radiotherapy, and chemotherapy were significantly associated with OS (Table 2). Further multivariate analysis showed that age, sex, race, tumor location, primary tumor size, pleural invasion, histological grade, TNM stage, primary tumor surgery, and chemotherapy were significantly associated with OS. Likewise, multivariate analysis identified that age, sex, race, tumor location, primary tumor size, pleural invasion, histological grade, TNM stage, primary tumor surgery, and chemotherapy were also significantly associated with CSS (Table 3). According to the multivariate results, two nomograms predicting the survival probability of 1- and 5-year OS (Figure 1) and CSS (Figure 2) were constructed with these independent variables.

To use the nomograms, each variable was first assigned to a specific score by the point scale at the top of the nomograms. Based on the sum of those scores, the point scale at the bottom of the nomograms was used to estimate the survival probability of one individual patient.

### 3.4. Nomogram Validation

**Figure 3:**
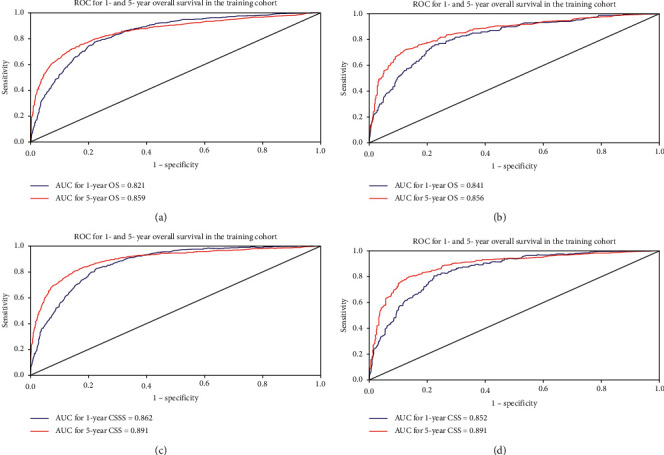
Receiver operating characteristic curves of the nomograms predicting OS and CSS in the training and validation cohorts. Receiver operating characteristic curves of 1- and 5-year OS in the training cohort (a) and the validation cohort (b); receiver operating characteristic curves of 1- and 5-year CSS in the training cohort (c) and the validation cohort (d).

**Figure 4:**
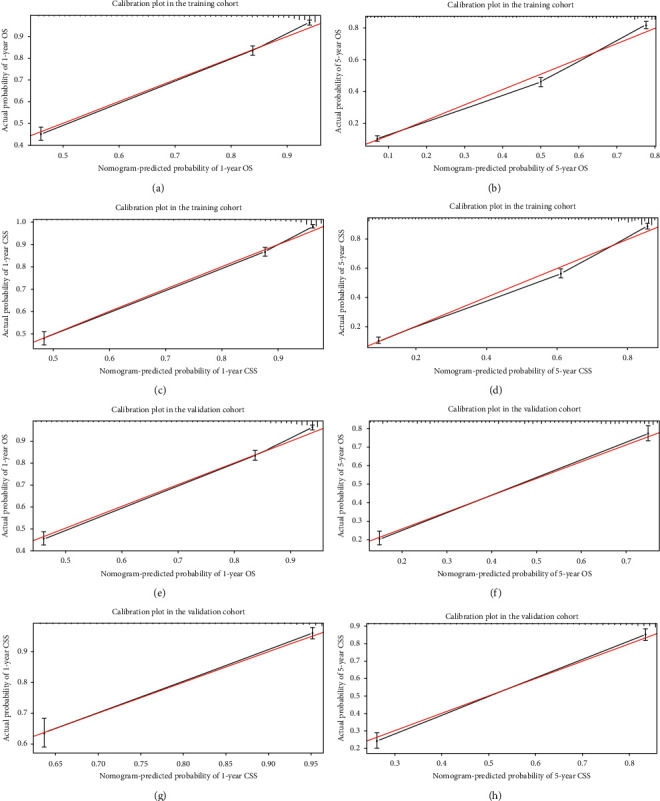
Calibration plots of the nomograms predicting OS and CSS in the training and validation cohorts. (a, b) Calibration plots of 1- and 5-year OS in the training cohort; (c, d) calibration plots of 1- and 5-year CSS in the training cohort; (e, f) calibration plots of 1- and 5-year OS in the validation cohort; (g, h) calibration plots of 1- and 5-year CSS in the validation cohort.

**Figure 5:**
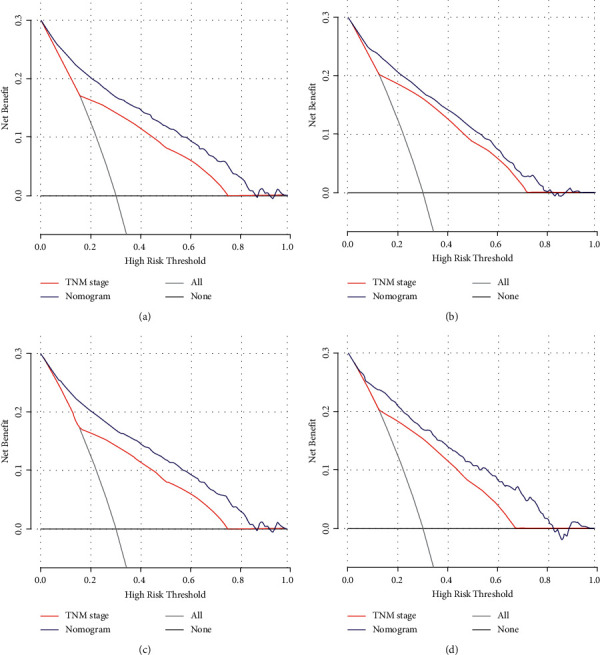
Decision curve analysis for the nomograms predicting OS and CSS. (a, b) Decision curve analysis of the nomogram for OS (a) and CSS (b) in the training cohort; (c, d) decision curve analysis of the nomogram for OS (c) and CSS (d) in the validation cohort.

Validation of the OS and CSS nomograms was performed in both the training and validation cohorts. The C-index values of the nomogram predicting OS and CSS were 0.786 (95% CI, 0.776–0.796) and 0.814 (95% CI, 0.804–0.824) in the training cohort, respectively. The *C*-index values of the nomogram predicting OS and CSS were 0.779 (95% CI, 0.757–0.801) and 0.807 (95% CI, 0.786–0.828) in the validation cohort, respectively. The sensitivity and specificity of predicting the prognosis of LPA were identified by ROC curves. As shown in Figure 3, the area under the curve (AUC) values of the nomogram predicting 1- and 5-year OS were 0.841 and 0.856, respectively, in the training cohort (Figure 3(a)); the AUC values of the nomogram predicting 1- and 5-year OS were 0.821 and 0.859, respectively, in the validation cohort (Figure 3(b)). While the AUC values of the nomogram predicting 1- and 5-year CSS were 0.862 and 0.891, respectively, in the training cohort (Figure 3(c)), the AUC values of the nomogram predicting 1- and 5-year CSS were 0.852 and 0.891, respectively, in the validation cohort (Figure 3(d)). Furthermore, calibration plots conducted using the training and validation cohorts both indicated that the OS and CSS nomograms demonstrated excellent agreement between the predicted and actual survival outcomes (Figures 4(a)–4(h)). In addition, the DCA results demonstrated that the nomograms showed better prognostic capacity than the TNM stage (Figures 5(a)–5(d)). As expected, when used to predict the survival outcomes of LPA patients, the nomogram constructed in this study was more accurate than a classic nomogram [9] previously established for overall NSCLC patients (Supplementary Figures S3A-S3B).

**Figure 6:**
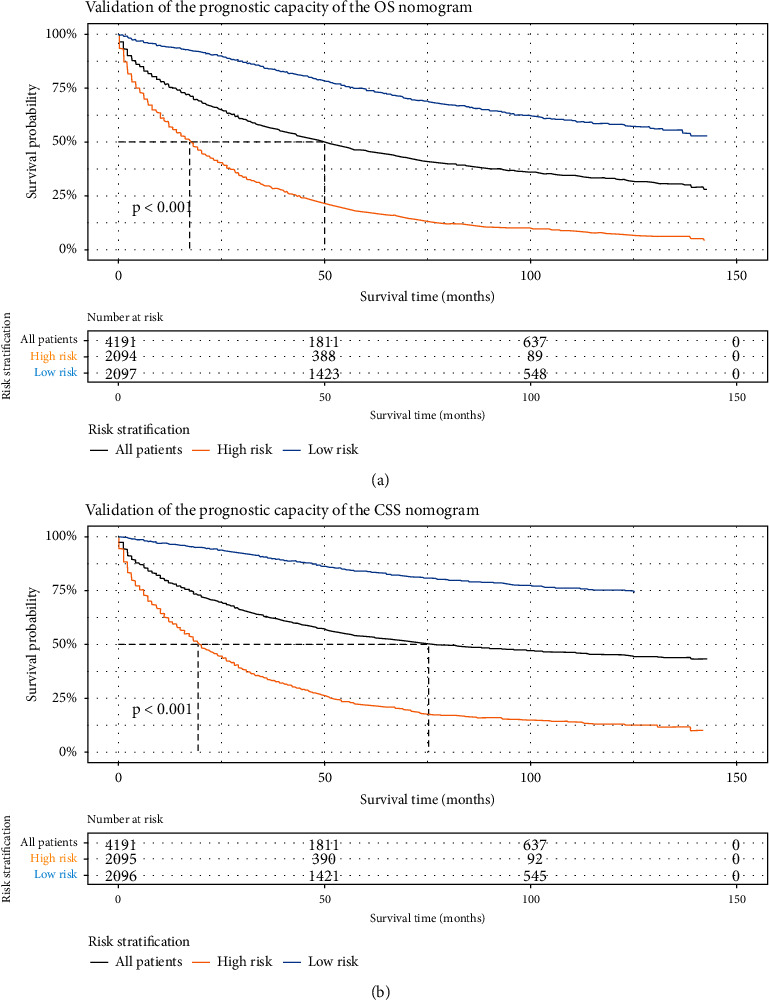
Kaplan–Meier curves of overall survival (a) and cancer-specific survival (b) for all patients with lepidic adenocarcinoma divided into two risk stratifications based on the scores calculated by the nomograms.

Furthermore, LPA patients were divided into two groups (“low risk” or “high risk”) based on the median total scores calculated by the nomograms. As shown in Figure 6(a), the Kaplan–Meier curves and log-rank tests suggested that the median OS of LPA patients in the high-risk group (17.0 months; 95% CI, 16.0–18.0 months) was significantly shorter than that in the low-risk group (not reached) (*P* < 0.001). Likewise, as shown in Figure 6(b), the median CSS of LPA patients in the high-risk group (19.0 months; 95% CI, 18.0–21.0 months) was significantly shorter than that in the low-risk group (not reached) (*P* < 0.001). Moreover, the median OS of all LPA patients was 50 months (95% CI, 47.0–53.0 months), and the median CSS of all LPA patients was 75 months (95% CI, 69.0–88.0 months) (Figure 6(a)–6(b)).

## 4. Discussion

Concise and accurate prognostic prediction models for patients with malignancy are essential for clinical decision-making and scientific research. Indeed, the TNM stage is the most widely used survival predictor for cancer patients. However, identifying more prognostic factors and a more individualized model will certainly improve the accuracy of clinical outcome prediction. In this study, we used the SEER database, a large-scale population-based cancer registry program, to explore the clinical characteristics of 4191 patients with LPA and identified the factors associated with distant and lymph node metastases in LPA patients. After that, we developed and validated accurate and personalized prognostic nomograms predicting the 1- and 5-year OS and CSS of patients with LPA.

The survival outcomes of LPA patients with poor prognostic factors were undesirable, and the median OS of advanced LPA patients was 20.1 months [10]. However, the prognosis of advanced LPA patients could be improved by appropriate treatments, including chemotherapy and EGFR tyrosine kinase inhibitors (TKIs) [10]. The 5-year disease-free survival of LPA patients after complete surgical resection was approximately 90% [11]. With the evaluation of the nomograms generated in our study, more aggressive treatments are recommended for high-risk patients with LPA, and appropriate shortening of the follow-up interval is encouraged to detect the occurrence of endpoint events as early as possible. For example, older, Black men with sizeable tumors and advanced TNM stages are recommended for frequent follow-up and more aggressive treatments, including primary tumor resection, when they meet the operational criteria.

Compared with other rare histologic subtypes of lung cancer, such as papillary adenocarcinoma [12] and carcinosarcoma [13], our results suggested that the incidence of LPA was much higher. Our results also indicated that LPA patients were more common in older age and females, which is consistent with previous studies [14, 15]. In addition, some clinicopathological features of LPA patients indicated a good prognosis, including smaller tumor size, fewer separate tumor nodules, less pleural invasion, and lower histological grade and stage. This is consistent with previous studies [16] and in line with the good prognosis of LPA [3, 14, 16]. Moreover, LPA possessed some characteristics differing from other histologic subtypes of invasive pulmonary ADC, such as being more common in nonsmokers or light smokers, having a preference for pulmonary peripheral location, and being false-negative in positron-emission tomographic scans [14, 17]. Like patients with NSCLC [18], we identified that variables including race, tumor size, separate tumor nodules, and histological grade were associated with distant and lymph node metastases in patients with LPA. Furthermore, asymptomatic at presentation or excessive airway secretion was more common in patients with LPA [19]. In the genetic alteration profiles, EGFR mutations occurred in approximately 50% of patients with LPA, which was significantly higher than other subtypes [5], especially mutations in exon 21 [19, 20]. However, KRAS mutations are much less common and account for approximately 10% of the LPA population [5]. Compared with other histologic subtypes, a lower rate of ALK rearrangement and a higher rate of RET rearrangement were reported [6, 21, 22].

Most studies supported patients with LPA had desirable survival outcomes compared with other subtypes of invasive pulmonary ADC. Surgery is still the superior option for LPA patients, whereas adjuvant chemotherapy, including oral fluoropyrimidines and platinum-based regimens, conferred no survival benefit on patients with LPA, regardless of the tumor stage [23, 24]. In patients with advanced LPA, studies have suggested that taxane-based chemotherapy and pemetrexed might be effective and well tolerated [25, 26]. With higher frequencies of EGFR mutations, EGFR-TKI therapy for advanced LPA demonstrated encouraging efficacy [10]. Nevertheless, due to the lower expression level of programmed cell death ligand 1, the efficacy of immune checkpoint inhibitors in patients with LPA may be poor [27–29]. Moreover, multiple studies suggested that a higher percentage of lepidic growth patterns were associated with a lower risk of recurrence, and invasive component size was a better predictor for survival than overall tumor diameter [17, 19, 30, 31]. Furthermore, no recurrence was observed in any of the 18 LPA patients with a maximum tumor diameter >3 cm but the maximum diameter of the invasive area <5 mm [32]. Therefore, Suzuki et al. [32] proposed that LPA with an invasion of 5 mm or less can be regarded as minimally invasive ADC even if the tumor is larger than 3 cm in diameter. Unsurprisingly, our results suggested that primary tumor surgery was a major prognostic factor of LPA patients following the TNM stage. In contrast, chemotherapy was far less important to the prognosis of LPA patients. Furthermore, our results suggested that radiotherapy had no significant effect on the survival outcomes of LPA patients. Regrettably, we could not explore the prognostic significance of chemotherapy regimens, targeted therapy, immunotherapy, or the diameter of the invasive area.

In this study, we identified that age, sex, race, tumor location, primary tumor size, pleural invasion, histological grade, TNM stage, primary tumor surgery, and chemotherapy were independently associated with OS and CSS in patients with LPA. Notably, few patients with histological grade IV LPA were included in this study. Therefore, the nomograms we constructed to predict the survival outcomes were not suitable for patients with histological grade IV LPA. Similar to previous studies, our results suggested that treatment, tumor size, and some demographic characteristics also had an impact on the prognosis of LPA patients, and we provided a statistical prediction tool that can incorporate and quantify the selected prognostic factors to estimate the survival outcome for an individual patient. Moreover, the nomograms were examined by C-index, ROC curves, calibration plots, and DCA curves, which demonstrated that the nomograms showed excellent agreement between the nomogram-predicted and actual survival outcomes of patients with LPA, as well as better prognostic capacity than TNM stage.

To date, this is the first time that the demographic and clinicopathological features, as well as the incidence of LPA, have been elucidated based on a large-scale population-based database. Meanwhile, this is the first nomogram predicting the survival outcomes of LPA patients, which could aid in the personalized prognostic evaluation and clinical decision-making. However, there were still some limitations in our study, although the nomograms demonstrated good accuracy and applicability. First, nomograms were constructed based on retrospective data, and prospective external validation is needed. Second, some critical information, such as the diameter of the invasive area in LPA, tumor biomarkers, chemotherapy regimen, targeted therapy, molecular pathology, and genetic tests, was absent in the database. Moreover, the TNM staging information provided by the database is the result of the 6th edition staging system, instead of the latest edition staging system. Therefore, we could not analyze those variables or improve the prognostic nomograms in our study. Third, the patients were almost all Americans, and the results might be different for other races. Such drawbacks are inherent to almost all retrospective population-based studies. However, the large sample size and the long follow-up duration of this study compensate to a great extent and provide comprehensive knowledge of LPA. Further prospective studies with more important information are needed for model improvement and independent validation.

## 5. Conclusion

In summary, we explored the clinical characteristics of LPA patients and developed nomograms predicting the OS and CSS of LPA patients individually. The nomograms showed good accuracy and applicability, which may aid in individualized prognostic prediction for LPA patients and clinical decision-making.

## Figures and Tables

**Table 1:** Characteristics of patients with LPA compared with lung ADC-NOS.

Characteristics	LPA *n* = 4191 (%)	ADC-NOS *n* = 95004 (%)	*P* value
Age, years			
0–69	1994 (47.6)	55203 (58.1)	<0.001
70–79	1438 (34.3)	26630 (28.0)	
≥80	759 (18.1)	13171 (13.9)	
Sex			
Male	1591 (38.0)	46856 (49.3)	<0.001
Female	2600 (62.0)	48148 (50.7)	
Race			
White	3372 (80.5)	74655 (78.6)	<0.001
Black	356 (8.5)	11469 (12.1)	
Others	463 (11.0)	8880 (9.3)	
Marital status			
Married	2460 (58.7)	52085 (54.8)	<0.001
Single	1731 (41.3)	42919 (45.2)	
Education			
High	2065 (49.3)	47790 (50.3)	0.197
Low	2126 (50.7)	47214 (49.7)	
Income			
High	2055 (49.0)	48741 (51.3)	0.004
Low	2136 (51.0)	46263 (48.7)	
Laterality			
Left	1620 (38.7)	36177 (38.1)	<0.001
Right	2447 (58.4)	54374 (57.2)	
Unknown	124 (3.0)	4453 (4.7)	
Lobe			
Upper	2195 (52.4)	51289 (54.0)	<0.001
Middle	199 (4.7)	4317 (4.5)	
Lower	1444 (34.5)	24035 (25.3)	
Unknown	353 (8.4)	15363 (16.2)	
Tumor size			
<3 cm	2229 (53.2)	31494 (33.2)	<0.001
≥3 and <5 cm	915 (21.8)	24387 (25.7)	
≥5 cm	530 (12.6)	22377 (23.6)	
Unknown	517 (12.3)	16746 (17.6)	
Separate tumor nodules			
Yes	361 (8.6)	15476 (16.3)	<0.001
No	1195 (28.5)	40383 (42.5)	
Unknown	2635 (62.9)	39145 (41.2)	
Pleural invasion			
Yes	117 (2.8)	5017 (5.3)	<0.001
No	714 (17.0)	11117 (11.7)	
Unknown	3360 (80.2)	78870 (83.0)	
Grade			
I	1636 (39.0)	6203 (6.5)	<0.001
II	1086 (25.9)	20984 (22.1)	
III	249 (5.9)	27993 (29.5)	
IV	7 (0.2)	606 (0.6)	
Unknown	1213 (28.9)	39218 (41.3)	
Stage			
I	2261 (53.9)	19370 (20.4)	<0.001
II	172 (4.1)	4007 (4.2)	
III	603 (14.4)	20127 (21.2)	
IV	1155 (27.6)	51500 (54.2)	
Primary tumor surgery			
Yes	2646 (63.1)	24886 (26.2)	<0.001
No/unknown	1545 (36.9)	70118 (73.8)	
Metastatic tumor surgery			
Yes	74 (1.8)	5499 (5.8)	<0.001
No/unknown	4117 (98.2)	89505 (94.2)	
Radiotherapy			
Yes	639 (15.2)	39072 (41.1)	<0.001
No/unknown	3552 (84.8)	55932 (58.9)	
Chemotherapy			
Yes	1226 (29.3)	46273 (48.7)	<0.001
No/unknown	2965 (70.7)	48731 (51.3)	

ADC: adenocarcinoma; LPA: lepidic adenocarcinoma; NOS: not otherwise specified.

**Table 2:** Univariate and multivariate analyses of OS in the training cohort of LPA patients (*n* = 3358).

Characteristics	Univariate analysis		Multivariate analysis	
	HR (95% CI)	*P*	HR (95% CI)	*P*
Age (70–79 vs. 0–69)	1.3 (1.18, 1.44)	<0.001	1.27 (1.15, 1.4)	<0.001
Age (≥80 vs. 0–69)	2.03 (1.81, 2.27)	<0.001	1.58 (1.4, 1.78)	<0.001
Sex (female vs. male)	0.59 (0.54, 0.65)	<0.001	0.64 (0.58, 0.7)	<0.001
Race (Black vs. White)	1.22 (1.05, 1.42)	0.01	1 (0.86, 1.17)	0.978
Race (Others vs. White)	0.8 (0.73, 0.87)	<0.001	0.81 (0.74, 0.88)	<0.001
Marital status (married vs. single)	1.18 (1.08, 1.29)	<0.001	1.05 (0.95, 1.16)	0.308
Education (low vs. high)	1.1 (1, 1.19)	0.038	1.02 (0.92, 1.12)	0.711
Income (low vs. high)	1.04 (0.95, 1.14)	0.421	—	—
Laterality (right vs. left)	1.22 (1, 1.49)	0.053	1.06 (0.87, 1.3)	0.565
Lobe (middle vs. upper)	1.25 (1.13, 1.37)	<0.001	1.14 (1.04, 1.26)	0.006
Lobe (lower vs. upper)	1.95 (1.75, 2.18)	<0.001	1.35 (1.2, 1.51)	<0.001
Tumor size (≥3 and <5 cm vs. <3 cm)	3.52 (3.1, 3.99)	<0.001	2.02 (1.77, 2.31)	<0.001
Tumor size (≥5 cm vs. <3 cm)	6.31 (5.57, 7.14)	<0.001	2.03 (1.75, 2.36)	<0.001
Separate tumor nodules (yes vs. no)	2.65 (2.1, 3.35)	<0.001	0.9 (0.71, 1.15)	0.406
Perineural invasion (yes vs. no/unknown)	3.15 (1.89, 5.24)	<0.001	2 (1.19, 3.36)	0.009
Grade (II vs. I)	1.2 (1.07, 1.35)	0.002	1.19 (1.06, 1.34)	0.004
Grade (III vs. I)	1.92 (1.61, 2.29)	<0.001	1.6 (1.33, 1.92)	<0.001
Grade (IV vs. I)	0.74 (0.18, 2.95)	0.665	0.65 (0.16, 2.63)	0.551
TNM stage (II vs. I)	2.36 (1.9, 2.93)	<0.001	2.18 (1.74, 2.72)	<0.001
TNM stage (III vs. I)	3.31 (2.92, 3.74)	<0.001	2.26 (1.97, 2.61)	<0.001
TNM stage (IV vs. I)	6 (5.41, 6.65)	<0.001	3.18 (2.76, 3.66)	<0.001
Primary tumor surgery (yes vs. no/unknown)	0.19 (0.17, 0.21)	<0.001	0.42 (0.37, 0.48)	<0.001
Metastatic tumor surgery (yes vs. no/unknown)	1.1 (0.82, 1.47)	0.512	-	-
Radiotherapy (yes vs. no/unknown)	2.16 (1.94, 2.41)	<0.001	1.11 (0.98, 1.25)	0.097
Chemotherapy (yes vs. no/unknown)	2.16 (1.97, 2.36)	<0.001	0.76 (0.68, 0.84)	<0.001

CI: confidence interval; HR: hazard ratio; LPA: lepidic adenocarcinoma; OS: overall survival.

**Table 3:** Univariate and multivariate analyses of CSS in the training cohort of LPA patients (*n* = 3358).

Variables	Univariate analysis		Multivariate analysis	
	HR (95% CI)	*P*	HR (95% CI)	*P*
Age (70–79 vs. 0–69)	1.17 (1.05, 1.31)	0.005	1.16 (1.03, 1.3)	0.011
Age (≥80 vs. 0–69)	1.6 (1.4, 1.83)	<0.001	1.25 (1.09, 1.44)	0.002
Sex (female vs. male)	0.6 (0.54, 0.66)	<0.001	0.67 (0.6, 0.74)	<0.001
Race (Black vs. White)	1.28 (1.08, 1.51)	0.005	0.99 (0.83, 1.18)	0.939
Race (Others vs. White)	0.84 (0.77, 0.93)	0.001	0.85 (0.77, 0.94)	0.002
Marital status (married vs. single)	1.21 (1.09, 1.33)	<0.001	1.04 (0.93, 1.16)	0.532
Education (low vs. high)	1.12 (1.01, 1.23)	0.028	1.05 (0.93, 1.17)	0.439
Income (low vs. high)	1.03 (0.93, 1.14)	0.581	—	—
Laterality (right vs. left)	1.23 (0.98, 1.54)	0.08	1.02 (0.81, 1.29)	0.867
Lobe (middle vs. upper)	1.26 (1.13, 1.41)	<0.001	1.13 (1.01, 1.26)	0.032
Lobe (lower vs. upper)	2.23 (1.96, 2.55)	<0.001	1.44 (1.26, 1.66)	<0.001
Tumor size (≥3 and <5 cm vs. <3 cm)	4.49 (3.9, 5.16)	<0.001	2.3 (1.98, 2.66)	<0.001
Tumor size (≥5 cm vs. <3 cm)	7.95 (6.92, 9.13)	<0.001	2.15 (1.82, 2.53)	<0.001
Separate tumor nodules (yes vs. no)	3.21 (2.5, 4.12)	<0.001	0.93 (0.71, 1.2)	0.559
Perineural invasion (yes vs. no/unknown)	3.95 (2.19, 7.11)	<0.001	2.15 (1.18, 3.91)	0.012
Grade (II vs. I)	1.24 (1.08, 1.42)	0.002	1.2 (1.05, 1.38)	0.009
Grade (III vs. I)	2.2 (1.81, 2.68)	<0.001	1.72 (1.41, 2.11)	<0.001
Grade (IV vs. I)	1.05 (0.26, 4.22)	0.943	0.83 (0.21, 3.37)	0.799
TNM stage (II vs. I)	3.45 (2.71, 4.39)	<0.001	3.05 (2.38, 3.92)	<0.001
TNM stage (III vs. I)	4.71 (4.07, 5.46)	<0.001	3.02 (2.56, 3.56)	<0.001
TNM stage (IV vs. I)	9.17 (8.11, 10.37)	<0.001	4.5 (3.82, 5.3)	<0.001
Primary tumor surgery (yes vs. no/unknown)	0.16 (0.14, 0.17)	<0.001	0.41 (0.35, 0.48)	<0.001
Metastatic tumor surgery (yes vs. no/unknown)	1.34 (1, 1.81)	0.054	-	-
Radiotherapy (yes vs. no/unknown)	2.24 (1.99, 2.53)	<0.001	1.07 (0.94, 1.22)	0.318
Chemotherapy (yes vs. no/unknown)	2.72 (2.46, 3.01)	<0.001	0.8 (0.72, 0.9)	<0.001

CI: confidence interval; CSS: cancer-specific survival; HR: hazard ratio; LPA: lepidic adenocarcinoma.

## Data Availability

The datasets generated and/or analyzed during this study are available in the Surveillance, Epidemiology, and End Results (SEER) database (https://seer.cancer.gov/). *R* code is available upon request.

## Supplementary Materials

Supplementary Materials Table S1. Characteristics of patients with LPA compared with lung papillary adenocarcinoma. Table S2. Characteristics of patients with LPA compared with lung solid adenocarcinoma. Table S3. Factors associated with distant metastasis in patients with lepidic adenocarcinoma (*n* = 4191). Table S4. Factors associated with lymph node metastasis in patients with lepidic adenocarcinoma (*n* = 4191). Table S5. Characteristics of patients with LPA in the training and validation cohorts. Figure S1. (A, B) Optimal cutoff value of age was identified by X-tail; (C) Kaplan–Meier curves of overall survival in three age subgroups (0–69, 70–79, and 80+ years old). Figure S2. Kaplan–Meier curves of overall survival (A) and cancer-specific survival (B) in the patients with lung ADC-NOS and LPA after propensity score matching. Abbreviations: ADC, adenocarcinoma; LPA, lepidic adenocarcinoma; NOS, not otherwise specified. Figure S3. Kaplan–Meier curves of overall survival (A) and cancer-specific survival (B) in the patients with lung papillary adenocarcinoma and LPA. Abbreviations: LPA, lepidic adenocarcinoma. Figure S4. Kaplan–Meier curves of overall survival (A) and cancer-specific survival (B) in the patients with lung solid adenocarcinoma and LPA. Abbreviations: LPA, lepidic adenocarcinoma. Figure S5. Receiver operating characteristic curves of the nomograms predicting OS and CSS in the training and validation cohorts using a classic nomogram previously established for overall NSCLC patients. Receiver operating characteristic curves of 1- and 5-year OS in the training cohort (A) and the validation cohort (B).
