# Supplementary material for: Characteristics and Prognostic Nomogram for Primary Lung Lepidic Adenocarcinoma
Source: Can Respir J. 2022 Aug 31;2022:3676547. doi: 10.1155/2022/3676547 (PMC9453021; doi:10.1155/2022/3676547)
Supplement: Supplementary Materials — Table S1. Characteristics of patients with LPA compared with lung papillary adenocarcinoma. Table S2. Characteristics of patients with LPA compared with lung solid adenocarcinoma. Table S3. Factors associated with distant metastasis in patients with lepidic adenocarcinoma (n = 4191). Table S4. Factors associated with lymph node metastasis in patients with lepidic adenocarcinoma (n = 4191). Table S5. Characteristics of patients with LPA in the training and validation cohorts. Figure S1. (A, B) Optimal cutoff value of age was identified by X-tail; (C) Kaplan–Meier curves of overall survival in three age subgroups (0–69, 70–79, and 80+ years old). Figure S2. Kaplan–Meier curves of overall survival (A) and cancer-specific survival (B) in the patients with lung ADC-NOS and LPA after propensity score matching. Abbreviations: ADC, adenocarcinoma; LPA, lepidic adenocarcinoma; NOS, not otherwise specified. Figure S3. Kaplan–Meier curves of overall survival (A) and cancer-specific survival (B) in the patients with lung papillary adenocarcinoma and LPA. Abbreviations: LPA, lepidic adenocarcinoma. Figure S4. Kaplan–Meier curves of overall survival (A) and cancer-specific survival (B) in the patients with lung solid adenocarcinoma and LPA. Abbreviations: LPA, lepidic adenocarcinoma. Figure S5. Receiver operating characteristic curves of the nomograms predicting OS and CSS in the training and validation cohorts using a classic nomogram previously established for overall NSCLC patients. Receiver operating characteristic curves of 1- and 5-year OS in the training cohort (A) and the validation cohort (B). [file 3676547.f1.docx]

**Supplementary materials**

Table S1. Characteristics of patients with LPA compared with lung papillary adenocarcinoma.

| **Characteristics** | **LPA** | **Papillary** | **P-value** |
| --- | --- | --- | --- |
|  | **n = 4191** | **n = 1545** |  |
|  | **Number (%)** | **Number (%)** |  |
| Age, years |  |  |  |
| 0-69 | 1994 (47.6) | 905 (58.6) | <0.001 |
| 70-79 | 1438 (34.3) | 458 (29.6) |  |
| >=80 | 759 (18.1) | 182 (11.8) |  |
| Sex |  |  |  |
| Male | 1591 (38.0) | 726 (47.0) | <0.001 |
| Female | 2600 (62.0) | 819 (53.0) |  |
| Race |  |  |  |
| White | 3372 (80.5) | 1140 (73.8) | <0.001 |
| Black | 356 (8.5) | 184 (11.9) |  |
| Others | 463 (11.0) | 221 (14.3) |  |
| Marital status |  |  |  |
| Married | 2460 (58.7) | 872 (56.4) | 0.132 |
| Single | 1731 (41.3) | 673 (43.6) |  |
| Laterality |  |  |  |
| Left | 1620 (38.7) | 594 (38.4) | 0.005 |
| Right | 2447 (58.4) | 878 (56.8) |  |
| Unknown | 124 (3.0) | 73 (4.7) |  |
| Lobe |  |  |  |
| Upper | 2195 (52.4) | 669 (43.3) | <0.001 |
| Middle | 199 (4.7) | 98 (6.3) |  |
| Lower | 1444 (34.5) | 563 (36.4) |  |
| Unknown | 353 (8.4) | 215 (13.9) |  |
| Tumor size |  |  |  |
| <3 cm | 2229 (53.2) | 661 (42.8) | <0.001 |
| >=3 & <5 cm | 915 (21.8) | 383 (24.8) |  |
| >=5 cm | 530 (12.6) | 287 (18.6) |  |
| Unknown | 517 (12.3) | 214 (13.9) |  |
| Separate tumor nodules |  |  |  |
| Yes | 361 (8.6) | 214 (13.9) | <0.001 |
| No | 1195 (28.5) | 709 (45.9) |  |
| Unknown | 2635 (62.9) | 622 (40.3) |  |
| Pleural invasion |  |  |  |
| Yes | 117 (2.8) | 157 (10.2) | <0.001 |
| No | 714 (17.0) | 371 (24.0) |  |
| Unknown | 3360 (80.2) | 1017 (65.8) |  |
| Grade |  |  |  |
| I | 1636 (39.0) | 307 (19.9) | <0.001 |
| II | 1086 (25.9) | 569 (36.8) |  |
| III | 249 (5.9) | 129 (8.3) |  |
| IV | 7 (0.2) | 8 (0.5) |  |
| Unknown | 1213 (28.9) | 532 (34.4) |  |
| Stage |  |  |  |
| I | 2261 (53.9) | 595 (38.5) | <0.001 |
| II | 172 (4.1) | 85 (5.5) |  |
| III | 603 (14.4) | 296 (19.2) |  |
| IV | 1155 (27.6) | 569 (36.8) |  |
| Primary tumor surgery |  |  |  |
| Yes | 2646 (63.1) | 869 (56.2) | <0.001 |
| No/unknown | 1545 (36.9) | 676 (43.8) |  |
| Metastatic tumor surgery |  |  |  |
| Yes | 74 (1.8) | 149 (9.6) | <0.001 |
| No/unknown | 4117 (98.2) | 1396 (90.4) |  |
| Radiotherapy |  |  |  |
| Yes | 639 (15.2) | 401 (26.0) | <0.001 |
| No/unknown | 3552 (84.8) | 1144 (74.0) |  |
| Chemotherapy |  |  |  |
| Yes | 1226 (29.3) | 657 (42.5) | <0.001 |
| No/unknown | 2965 (70.7) | 888 (57.5) |  |

Abbreviations: LPA, lepidic adenocarcinoma.

Table S2. Characteristics of patients with LPA compared with lung solid adenocarcinoma.

| **Characteristics** | **LPA** | **Solid** | **P-value** |
| --- | --- | --- | --- |
|  | **n = 4191** | **n = 163** |  |
|  | **Number (%)** | **Number (%)** |  |
| Age, years |  |  |  |
| 0-69 | 1994 (47.6) | 112 (68.7) | <0.001 |
| 70-79 | 1438 (34.3) | 38 (23.3) |  |
| >=80 | 759 (18.1) | 13 (8.0) |  |
| Sex |  |  |  |
| Male | 1591 (38.0) | 98 (60.1) | <0.001 |
| Female | 2600 (62.0) | 65 (39.9) |  |
| Race |  |  |  |
| White | 3372 (80.5) | 139 (85.3) | 0.084 |
| Black | 356 (8.5) | 15 (9.2) |  |
| Others | 463 (11.0) | 9 (5.5) |  |
| Marital status |  |  |  |
| Married | 2460 (58.7) | 94 (57.7) | 0.857 |
| Single | 1731 (41.3) | 69 (42.3) |  |
| Laterality |  |  |  |
| Left | 1620 (38.7) | 58 (35.6) | 0.65 |
| Right | 2447 (58.4) | 101 (62.0) |  |
| Unknown | 124 (3.0) | 4 (2.5) |  |
| Lobe |  |  |  |
| Upper | 2195 (52.4) | 103 (63.2) | 0.002 |
| Middle | 199 (4.7) | 13 (8.0) |  |
| Lower | 1444 (34.5) | 36 (22.1) |  |
| Unknown | 353 (8.4) | 11 (6.7) |  |
| Tumor size |  |  |  |
| <3 cm | 2229 (53.2) | 77 (47.2) | <0.001 |
| >=3 & <5 cm | 915 (21.8) | 49 (30.1) |  |
| >=5 cm | 530 (12.6) | 31 (19.0) |  |
| Unknown | 517 (12.3) | 6 (3.7) |  |
| Separate tumor nodules |  |  |  |
| Yes | 361 (8.6) | 16 (9.8) | <0.001 |
| No | 1195 (28.5) | 128 (78.5) |  |
| Unknown | 2635 (62.9) | 19 (11.7) |  |
| Pleural invasion |  |  |  |
| Yes | 117 (2.8) | 46 (28.2) | <0.001 |
| No | 714 (17.0) | 69 (42.3) |  |
| Unknown | 3360 (80.2) | 48 (29.4) |  |
| Grade |  |  |  |
| I | 1636 (39.0) | 1 (0.6) | <0.001 |
| II | 1086 (25.9) | 21 (12.9) |  |
| III | 249 (5.9) | 126 (77.3) |  |
| IV | 7 (0.2) | 4 (2.5) |  |
| Unknown | 1213 (28.9) | 11 (6.7) |  |
| Stage |  |  |  |
| I | 2261 (53.9) | 74 (45.4) | <0.001 |
| II | 172 (4.1) | 28 (17.2) |  |
| III | 603 (14.4) | 34 (20.9) |  |
| IV | 1155 (27.6) | 27 (16.6) |  |
| Primary tumor surgery |  |  |  |
| Yes | 2646 (63.1) | 136 (83.4) | <0.001 |
| No/unknown | 1545 (36.9) | 27 (16.6) |  |
| Metastatic tumor surgery |  |  |  |
| Yes | 74 (1.8) | 11 (6.7) | <0.001 |
| No/unknown | 4117 (98.2) | 152 (93.3) |  |
| Radiotherapy |  |  |  |
| Yes | 639 (15.2) | 45 (27.6) | <0.001 |
| No/unknown | 3552 (84.8) | 118 (72.4) |  |
| Chemotherapy |  |  |  |
| Yes | 1226 (29.3) | 78 (47.9) | <0.001 |
| No/unknown | 2965 (70.7) | 85 (52.1) |  |

Abbreviations: LPA, lepidic adenocarcinoma.

Table S3. Factors associated with distant metastasis in patients with lepidic adenocarcinoma (n = 4191).

| **Characteristics** | **Univariate analysis** | | **Multivariate analysis** | |
| --- | --- | --- | --- | --- |
|  | **OR (95% CI)** | **P** | **OR (95% CI)** | **P** |
| Age (70-79 vs. 0-69) | 0.96(0.83,1.12) | 0.608 | - | - |
| Age (≥80 vs. 0-69) | 1.06(0.88,1.27) | 0.569 | - | - |
| Sex (Female vs. Male) | 0.8(0.69,0.91) | 0.001 | 1.16(0.97,1.38) | 0.097 |
| Race (Black vs. White) | 1.46(1.16,1.85) | 0.001 | 1.31(0.97,1.75) | 0.074 |
| Race (Others vs. White) | 1.46(1.19,1.8) | <0.001 | 1.58(1.22,2.05) | 0.001 |
| Laterality (Right vs. Left) | 1.01(0.87,1.16) | 0.914 | - | - |
| Lobe (Middle vs. Upper) | 1.25(0.9,1.75) | 0.183 | 1.34(0.91,1.97) | 0.14 |
| Lobe (Lower vs. Upper) | 1.2(1.03,1.41) | 0.02 | 1.06(0.88,1.27) | 0.559 |
| Tumor size (≥3 & <5 cm vs. <3 cm) | 2.47(2.05,2.98) | <0.001 | 2.18(1.77,2.69) | <0.001 |
| Tumor size (≥5 cm vs. <3 cm) | 4.01(3.25,4.96) | <0.001 | 3.27(2.57,4.16) | <0.001 |
| Separate tumor nodules (Yes vs. No) | 21.68(16.15,29.1) | <0.001 | 20.87(14.52,30.01) | <0.001 |
| Pleural invasion (Yes vs. No) | 3.17(1.9,5.29) | <0.001 | 1.54(0.78,3.04) | 0.209 |
| Grade (II vs. I) | 0.91(0.75,1.1) | 0.326 | 0.87(0.69,1.09) | 0.228 |
| Grade (III vs. I) | 1.6(1.19,2.15) | 0.002 | 1.44(1.02,2.04) | 0.041 |
| Grade (IV vs. I) | 0.64(0.08,5.35) | 0.683 | 0.15(0.01,2.56) | 0.189 |

Abbreviations: CI, confidence interval; OR, odds ratio.

Table S4. Factors associated with lymph node metastasis in patients with lepidic adenocarcinoma (n = 4191).

| **Characteristics** | **Univariate analysis** | | **Multivariate analysis** | |
| --- | --- | --- | --- | --- |
|  | **OR (95% CI)** | **P** | **OR (95% CI)** | **P** |
| Age (70-79 vs. 0-69) | 0.81(0.69,0.95) | 0.011 | 0.77(0.65,0.91) | 0.002 |
| Age (≥80 vs. 0-69) | 0.83(0.68,1.01) | 0.061 | 0.68(0.55,0.84) | <0.001 |
| Sex (Female vs. Male) | 0.7(0.61,0.81) | <0.001 | 0.83(0.71,0.97) | 0.018 |
| Race (Black vs. White) | 1.19(0.93,1.53) | 0.17 | 1.02(0.78,1.33) | 0.9 |
| Race (Others vs. White) | 1.42(1.15,1.76) | 0.001 | 1.39(1.1,1.75) | 0.005 |
| Laterality (Right vs. Left) | 1.08(0.93,1.25) | 0.315 | - | - |
| Lobe (Middle vs. Upper) | 1.2(0.86,1.67) | 0.273 | - | - |
| Lobe (Lower vs. Upper) | 1.03(0.88,1.2) | 0.73 | - | - |
| Tumor size (≥3 & <5 cm vs. <3 cm) | 2.45(2.04,2.94) | <0.001 | 2.18(1.8,2.64) | <0.001 |
| Tumor size (≥5 cm vs. <3 cm) | 4.13(3.35,5.09) | <0.001 | 3.55(2.86,4.42) | <0.001 |
| Separate tumor nodules (Yes vs. No) | 2.21(1.7,2.88) | <0.001 | 1.32(0.99,1.77) | 0.06 |
| Pleural invasion (Yes vs. No) | 3.35(2.05,5.47) | <0.001 | 2.18(1.31,3.65) | 0.003 |
| Grade (II vs. I) | 1.81(1.49,2.19) | <0.001 | 1.68(1.37,2.05) | <0.001 |
| Grade (III vs. I) | 3.23(2.42,4.32) | <0.001 | 2.76(2.03,3.75) | <0.001 |
| Grade (IV vs. I) | 4.14(0.92,18.6) | 0.064 | 2.6(0.55,12.2) | 0.225 |

Abbreviations: CI, confidence interval; OR, odds ratio.

Table S5. Characteristics of patients with LPA in the training and validation cohorts.

| **Characteristics** | **Training cohort** | **Validation cohort** | **P-value** |
| --- | --- | --- | --- |
|  | **n = 3358** | **n = 833** |  |
|  | **Number (%)** | **Number (%)** |  |
| Age, years |  |  |  |
| 0-69 | 1629 (48.5) | 365 (43.8) | 0.02 |
| 70-79 | 1144 (34.1) | 294 (35.3) |  |
| >=80 | 585 (17.4) | 174 (20.9) |  |
| Sex |  |  |  |
| Male | 1285 (38.3) | 306 (36.7) | 0.438 |
| Female | 2073 (61.7) | 527 (63.3) |  |
| Race |  |  |  |
| White | 2737 (81.5) | 635 (76.2) | 0.002 |
| Black | 276 (8.2) | 80 (9.6) |  |
| Other | 345 (10.3) | 118 (14.2) |  |
| Marital status |  |  |  |
| Married | 1969 (58.6) | 491 (58.9) | 0.903 |
| Single | 1389 (41.4) | 342 (41.1) |  |
| Education |  |  |  |
| Low | 1680 (50.0) | 385 (46.2) | 0.054 |
| High | 1678 (50.0) | 448 (53.8) |  |
| Income |  |  |  |
| Low | 1664 (49.6) | 391 (46.9) | 0.189 |
| High | 1694 (50.4) | 442 (53.1) |  |
| Laterality |  |  |  |
| Left | 1317 (39.2) | 303 (36.4) | 0.234 |
| Right | 1946 (58.0) | 501 (60.1) |  |
| Unknown | 95 (2.8) | 29 (3.5) |  |
| Lobe |  |  |  |
| Upper | 1753 (52.2) | 442 (53.1) | 0.431 |
| Middle | 166 (4.9) | 33 (4.0) |  |
| Lower | 1164 (34.7) | 280 (33.6) |  |
| Unknown | 275 (8.2) | 78 (9.4) |  |
| Tumor Size |  |  |  |
| <3 cm | 1752 (52.2) | 477 (57.3) | 0.051 |
| >=3 & <5 cm | 757 (22.5) | 158 (19.0) |  |
| >=5 cm | 430 (12.8) | 100 (12.0) |  |
| Unknown | 419 (12.5) | 98 (11.8) |  |
| Separate tumor nodules |  |  |  |
| Yes | 165 (4.9) | 196 (23.5) | <0.001 |
| No | 587 (17.5) | 608 (73.0) |  |
| Unknown | 2606 (77.6) | 29 (3.5) |  |
| Pleural invasion |  |  | <0.001 |
| Yes | 74 (2.4) | 36 (3.7) |  |
| No | 406 (13.1) | 271 (27.7) |  |
| Unknown | 2628 (84.6) | 672 (68.6) |  |
| Grade |  |  | 0.004 |
| I | 1170 (37.6) | 422 (43.1) |  |
| II | 828 (26.6) | 241 (24.6) |  |
| III | 203 (6.5) | 41 (4.2) |  |
| IV | 4 (0.1) | 3 (0.3) |  |
| Unknown | 903 (29.1) | 272 (27.8) |  |
| Stage |  |  | 0.309 |
| I | 1672 (53.8) | 535 (54.6) |  |
| II | 132 (4.2) | 35 (3.6) |  |
| III | 459 (14.8) | 126 (12.9) |  |
| IV | 845 (27.2) | 283 (28.9) |  |
| Primary tumor surgery |  |  | 0.844 |
| Yes | 1978 (63.6) | 619 (63.2) |  |
| No/unknown | 1130 (36.4) | 360 (36.8) |  |
| Metastatic tumor surgery |  |  | 0.375 |
| Yes | 60 (1.9) | 14 (1.4) |  |
| No/unknown | 3048 (98.1) | 965 (98.6) |  |
| Radiotherapy |  |  | 0.75 |
| Yes | 469 (15.1) | 143 (14.6) |  |
| No/unknown | 2639 (84.9) | 836 (85.4) |  |
| Chemotherapy |  |  | 0.136 |
| Yes | 927 (29.8) | 267 (27.3) |  |
| No/unknown | 2181 (70.2) | 712 (72.7) |  |

Abbreviations: LPA, lepidic adenocarcinoma.


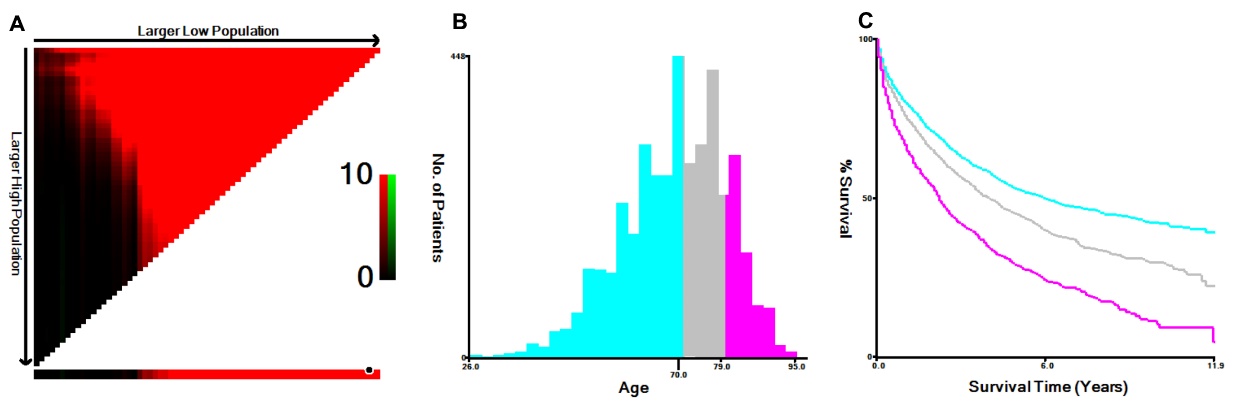


Figure S1. (A, B) The optimal cutoff value of age was identified by X-tail; (C) the Kaplan–Meier curves of overall survival in three age subgroups (0–69, 70–79, and 80+ years old).


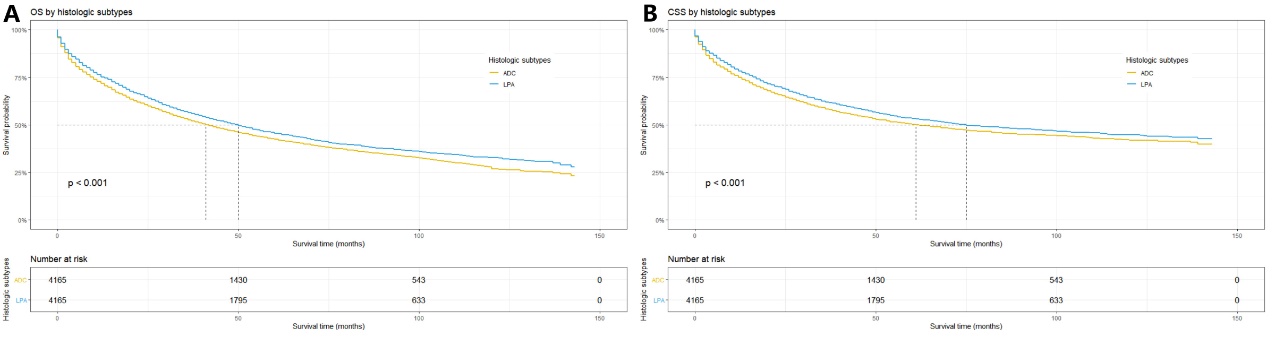


Figure S2. Kaplan–Meier curves of overall survival (A) and cancer-specific survival (B) in the patients with lung ADC-NOS and LPA after propensity score matching.

Abbreviations: ADC, adenocarcinoma; LPA, lepidic adenocarcinoma; NOS, not otherwise specified.


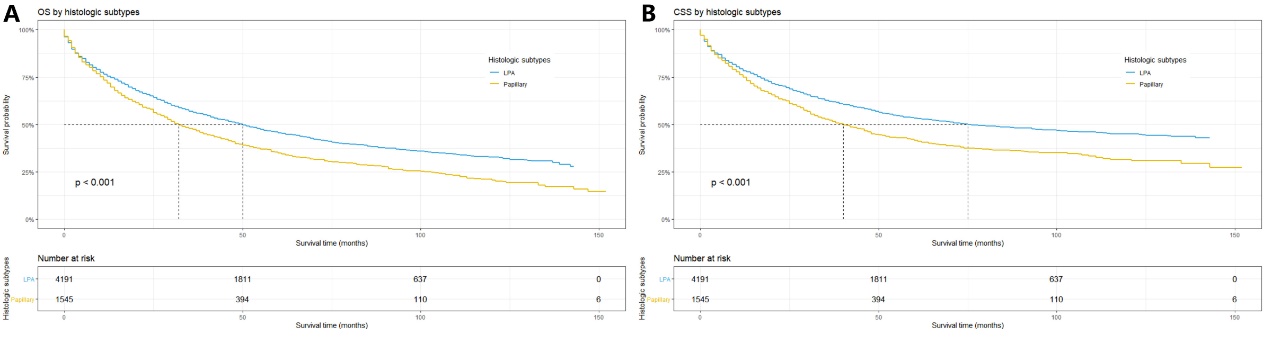


Figure S3. Kaplan–Meier curves of overall survival (A) and cancer-specific survival (B) in the patients with lung papillary adenocarcinoma and LPA.

Abbreviations: LPA, lepidic adenocarcinoma.


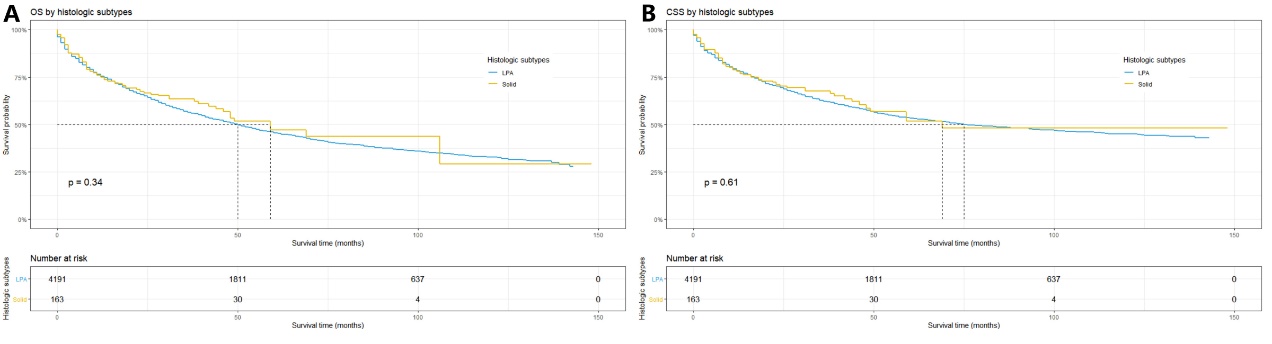


Figure S4. Kaplan–Meier curves of overall survival (A) and cancer-specific survival (B) in the patients with lung solid adenocarcinoma and LPA.

Abbreviations: LPA, lepidic adenocarcinoma.


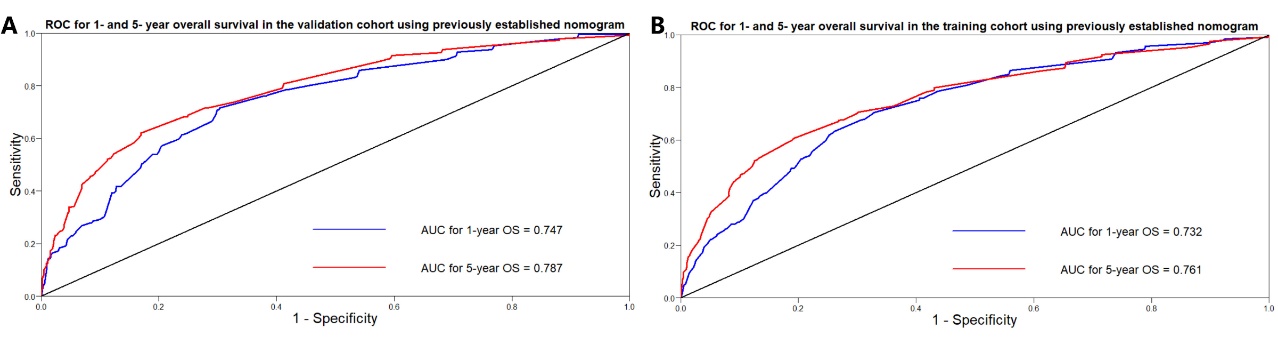
Figure S5. Receiver operating characteristic curves of the nomograms predicting OS and CSS in the training and validation cohorts using a classic nomogram previously established for overall NSCLC patients. Receiver operating characteristic curves of 1- and 5-year OS in the training cohort (A) and the validation cohort (B).
